# Expiratory time constants in mechanically ventilated patients: rethinking the old concept—a narrative review

**DOI:** 10.1186/s40635-025-00745-9

**Published:** 2025-03-26

**Authors:** Filip Depta, Richard H. Kallet, Michael A. Gentile, Elias N. Baedorf Kassis

**Affiliations:** 1 Department of Critical Care, East Slovak Institute for Cardiovascular Diseases and Faculty of Medicine, Pavol Jozef Šafárik University, Košice, Slovakia; 2 Respiratory Care Services, Department of Anesthesia and Perioperative Care, San Francisco General Hospital, San Francisco, CA USA; 3 Department of Anesthesiology, Duke University Medical Center Durham, Durham, NC USA; 4 Beth Israel Deaconess Medical Center and Harvard Medical School, Boston, MA USA

**Keywords:** Expiratory time constant, Mechanical ventilation, Flow-volume waveform, Positive end-expiratory pressure, Review

## Abstract

The expiratory time constant (RC_EXP_) plays an important role in understanding the mechanical properties of the respiratory system in patients receiving mechanical ventilation. Initially conceived as a tool to illustrate nonlinearity in lung emptying, RC_EXP_ has transitioned from a theoretical concept to a clinically relevant parameter, particularly within the realm of intelligent ventilation strategies. This narrative review explores the historical development of RC_EXP_, starting with its foundational definition based on fixed values of respiratory system resistance and compliance (i.e., the single-compartmental model). This early approach to RC_EXP_ largely overlooked the intricate viscoelastic characteristics of the lungs. The inherent limitations of this simplified model are discussed. The review then shifts its focus to clinical evidence describing the severity of deviations in RC_EXP_ from the ‘‘ideal’’ state in both acute lung injury and obstructive lung disease. This includes an analysis of which portions of the expiratory phase are most affected and how adjustments in tidal volume and positive end-expiratory pressure can potentially improve the homogeneity of lung emptying. The review concludes with a discussion of the clinical applications of RC_EXP_ and proposes future directions for its integration into ventilator management.

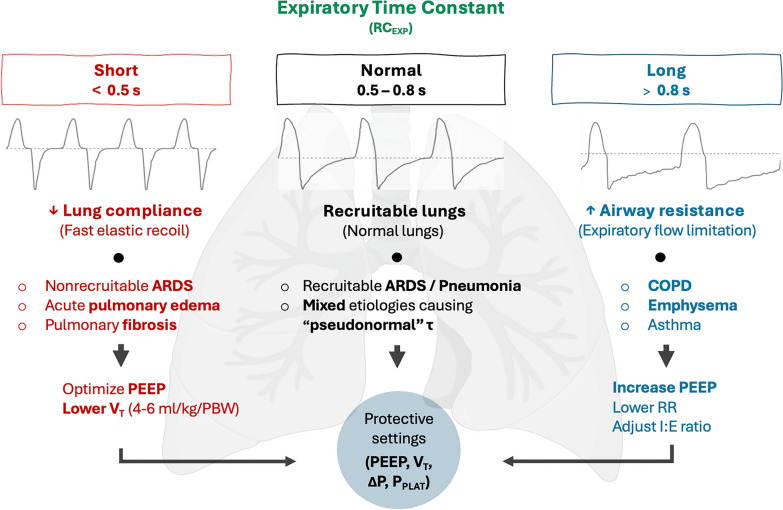

## Introduction

Recent advances in mechanical ventilator monitoring allow for the assessment of advanced pulmonary mechanics, which can evaluate the impact of ventilator adjustments to optimize patient care. Of particular interest is the ability to measure expiratory time constant (RC_EXP_) to enhance adjustments in tidal volume (V_T_), positive end-expiratory pressure (PEEP), respiratory rate (RR), and, consequently, minute ventilation. This holds true mostly using closed-loop system adaptive modes of ventilation [1]. The goal is to minimize dynamic hyperinflation and its impact on both lung stress–strain and hemodynamic impairment from intrinsic PEEP. Although the concept of pulmonary time constants dates back to the 1950s, perhaps its most significant application was introduced 20 years ago with the advent of closed-loop intelligent ventilation platforms. [2].

This narrative review provides a brief orientation and history of this concept. It then defines expiratory time constant (RC_EXP_), including reference values and findings of different methods used to obtain it. The review also considers the behavior of RC_EXP_ given different lung pathologies, including its conceptual limitations in light of these clinical investigations and the implications of these findings for patient management.

### Brief history of pulmonary time constants

A time constant describes the equilibration characteristics of pressure, flow, and volume over time between two points in the patient–ventilator circuit. This theoretical model was based upon the simple premise of a constant pressure generator connected to a single compartment with fixed resistance and a compliance component. In other words, it describes passive pressure control ventilation, whereby a fixed airway pressure produces an exponential curve for changes in pressure, flow, and volume changes between the proximal airway and the alveoli [3, 4]. Although the time constant can be determined for both inspiration (i.e., the inspiratory time constant is essentially influenced by ventilator settings during the inspiratory phase) and exhalation, only the RC_EXP_ is a result of passive elastic recoil of the lung and the chest wall. This distinction is important for understanding the respiratory mechanics of passive exhalation; therefore, this review will focus solely on RC_EXP_.

The distinct shape of any exponential curve is based upon two parameters that remain constant for any particular condition. First is the *“amplitude constant,”* which signifies the magnitude of the applied pressure driving ventilation at breath initiation (start of inspiration) or breath termination (start of exhalation). Second, the *“time constant”* represents the magnitude of inspiratory or expiratory volume with corresponding flow. The term *expiratory time constant* may be misleading and confusing as it should refer to a different portion of exhaled flow (and thus volume) during an exponential process per the same amount of time (hence the term constant). The time-constant concept, originally designed to explain the physiology of lung emptying, has major limitations when applied to patients with healthy or heterogeneous diseased lungs under mechanical ventilation. In this context, exhalation is influenced by components that introduce additional resistance, such as the endotracheal tube, the ventilation circuit's resistance, and the resistance of the mechanical ventilator expiratory valve. As a result of these complexities, the term expiratory time “constant", which was initially meant to indicate a consistent duration of defined parts of passive exhalation, does not accurately reflect the actual dynamics of airflow during exhalation in real-life clinical scenarios [5, 6].

Previous literature uses varying abbreviations for the expiratory time constant, often called ‘‘RC_EXP_’’ (the product of resistance and compliance), while the measured value is typically denoted by the Greek letter “tau” (*τ*). This review uses both terms interchangeably.

### Definition and physiological basis of RC_EXP_

RC_EXP_ is a product of respiratory system compliance (C_RS_) and respiratory system resistance (R_RS_) with the resulting unit in seconds (Eq. 1) [7]:

1 $$ {\text{RC}}_{{{\text{EXP}}}} = {\text{C}}_{{{\text{RS}}}} { } \times {\text{ R}}_{{{\text{RS}}}} = \frac{{{\mathbf{V}}_{{\mathbf{T}}} { }\left( {\text{L}} \right)}}{{{\mathbf{\Delta P}}{ }\left( {{\text{cmH}}2{\text{O}}} \right)}}{ } \times { }\frac{{{\mathbf{\Delta P}}{ }\left( {{\text{cmH}}2{\text{O}}} \right)}}{{{\mathbf{V^{\prime}}}\left( {{\text{L}}/{\text{s}}} \right)}} = \frac{{{\mathbf{V}}_{{\mathbf{T}}} { }\left( {\text{L}} \right)}}{{{\mathbf{V^{\prime}}}\left( {{\text{L}}/{\text{s}}} \right)}} = {\text{L }} \times \frac{{\text{s}}}{{\text{L}}} = {\text{s}} $$

*where ΔP is the difference between plateau pressure and PEEP, pressure,* *V*’ *is expiratory flow, L—liters, s—seconds.*

Exhalation is a mostly passive process utilizing the stored elastic energy from respiratory distension from inspiration to drive expiratory flow following the expected exponential decay. RC_EXP_ represents the time when such exponential change would be complete if the rate of change were maintained at its initial level rather than decreasing [4]. In other words, it is the linear expression of a distinctly nonlinear phenomenon. Expressing *τ* as “incomplete” change (I) uses the base of natural logarithms (e) such that I/e = 100% ÷ 2.718 = 37%, and thereby completed change at 1τ would be 63%; at 2τ it would be calculated as 37% ÷ 2.718 or ~ 14% incomplete and ~ 86% complete, etc. Following such exponential kinetics, 63%, 86%, 95%, 98%, and 99% of V_T_ are exhaled by one, two, three, four, and five τ, respectively [7, 8]**.**

Interestingly, only *“one”* or the *“first”* RC_EXP_ appears in most clinical studies, leaving the duration of the rest of the exhalation without numeric expression.

### Reference values of RC_EXP_

The average reference values for adult patients with preserved lung function on invasive mechanical ventilation range between 0.5 and 0.8s, with a mean value of around 0.6s (Table 1) [2, 9–16]. However, coexisting pathologies may render RC_EXP_ to appear normal (i.e., RC_EXP_ pseudo-normalization). This may result from conditions associated with high R_RS_ but low C_RS_. These values should be taken in context with set inspiratory variables, most notably V_T_ and PEEP. Even in healthy postoperative patients without known previous lung disease, RC_EXP_ slightly differs, depending on the applied methodology to obtain RC_EXP_ [9–12, 14].

**Table 1:** Typical range of time constants in passive intubated patients with various disease states

Short RC_EXP_ < 0.5 s	Normal RC_EXP_ 0.5–0.8 s	Long RC_EXP_ > 0.8 s
Nonrecruitable (stiff) ARDS	Normal lungs	COPD
Fibrosis	Recruitable lungs	Asthma
Acute pulmonary edema	Mixed conditions*	Emphysema
Kyphoscoliosis		Chronic bronchitis
Chest wall stiffness		Bronchospasm

^*^Coexisting (mixed) pathologies can have pseudo-normal RC_EXP_ due to various combinations of R_RS_ and C_RS_. ARDS—acute respiratory distress syndrome, *COPD* chronic obstructive pulmonary disease, *RC*_*EXP*_ expiratory time constant

The importance of this concept is the following: [1] when R_RS_ is held constant, equilibration time for flow, volume, and pressure is directly proportional to C_RS_ (i.e., as C_RS_ decreases with a stiffer/smaller lung, equilibration time also decreases), and [2] when C_RS_ is held constant, equilibration also is directly proportional to R_RS_ (i.e., as R_RS_ increases, equilibration time is prolonged). In addition, the actual time needed for equilibration depends upon the magnitude of V_T_ or associated driving pressure (ΔP) and PEEP. This provides the context in which time-constant values should interpreted. Table 2 summarizes the major RC_EXP_ determinants in two common clinical entities—ARDS and COPD.

**Table 2:** Major RC_EXP_ determinants in two common clinical entities—ARDS and COPD

RC_EXP_ determinant	ARDS	COPD
ETT [6]	The ETT represents a major resistance in the patient–ventilator system	Patients have high intrinsic resistance (due to diseased narrowed airways). Therefore, ETT plays less important role in total resistance
PEEP [15, 26]	PEEP below the lower inflection point and inappropriately high PEEP causing overdistension lead to shorter RC_EXP_ compared to RC_EXP_ values within the recruitable range	PEEP is the single most important determinant to decrease the global RC_EXP_ and promote exhalation and minimize air trapping
V_T_ [8]	With recruitable lungs, higher V_T_ leads to longer RC_EXP_, but must be considered alongside PEEP	In view of high intrinsic airway resistance, V_T_ becomes less important determinant of RC_EXP_ compared to PEEP
RR [16]	RR and RC_EXP_ are inversely related; higher RC_EXP_ needs lower RR, while lower RC_EXP_ allows for higher RR. In stiff, nonrecruitable lungs, RR affects RC_EXP_ minimally	In COPD patients, lower RR is needed, but reducing RR affects expiratory resistance less than increasing PEEP
I:E ratio	In general, longer RC_EXP_ require higher I: E ratios. Stiff ARDS lungs (due to the short RC_EXP_ and rapid emptying) is practically unaffected by I:E ratio modification	COPD patients need higher I:E ratios to promote exhalation, but in severe COPD, the effect is minimal due to high intrinsic airway resistance

*ARDS* acute respiratory distress syndrome, *COPD* chronic obstructive pulmonary disease, *ETT* endotracheal tube, *I: E ratio* inspiratory to expiratory ratio, *PEEP* positive end-expiratory pressure, *V*_*T*_ tidal volume, *RR* respiratory rate

As mentioned previously, by definition, a near-full exhalation should be completed in three-time constants (i.e., 95% of V_T_) [7]. However, due to the physics of pressure and flow equilibration over time, 95% of V_T_ is exhaled sooner. The first, second, and third RC_EXP_ are not equal but progressively shorter, with mean **τ**_**1**_, **τ**_**2**_, and **τ**_**3**_ of 0.6s, 0.4s, and 0.3s, respectively, regardless of whether pressure-controlled ventilation (i.e., variable flow) or volume-controlled (i.e., constant flow) ventilation was used [14]. The phenomenon described arises from the rapid decrease in airway pressure coupled with a more gradual reduction in airflow during the early phase of exhalation. As a result, the significant resistance introduced by the endotracheal tube (ETT) produces longer RC_EXP_ during early exhalation but progressively declining RC_EXP_ during later parts of exhalation, where ETT-induced resistance applies to a much lesser degree.

The concept of time constant deviates from reality, because both R_RS_ and C_RS_ change during either phase of ventilation. This is especially relevant during passive exhalation, because ΔP decreases with lung volume, while R_RS_ increases (i.e., decreased in radial traction supporting peripheral airway patency). In addition, depending on the PEEP, C_RS_ may change during exhalation resulting in progressive alveolar collapse. Therefore, using the single exponential model using single values or C_RS_ and R_RS_ to demonstrate RC_EXP_ provides only an overall rough approximation of what is observed clinically [17].

### Methods to determine RC_EXP_

In the context of a single-compartmental model, two linear equations have been proposed to calculate RC_EXP_. The first method involves calculating the product of C_RS_ and expiratory resistance [7, 18]. The second method examines the change in V_T_ during exhalation with the corresponding change in expiratory flow rate. The resulting RC_EXP_ is determined by dividing V_T_ by the peak expiratory flow (PEF). This method was initially described by Marini and later refined by Brunner (Eq. 3), who included a correction factor to account for incomplete exhalation [19]. The resulting RC_EXP_ using these two methods are essentially the same, because the product of C_RS_ and R_RS_ equals the fraction of V_T_ over PEF (Eq. 1):

2 $${RC}_{EXP}={C}_{RS} {\text{x }R}_{E}$$

where *R*_E_ is expiratory airway resistance

3 $${RC}_{EXP}=\frac{{V}_{T}}{PEF}$$

The nonlinearity of the flow/volume waveform (*V’*/*V* curve) during passive exhalation indicates that expiratory flow starts at a relatively high rate in the early phase (i.e., PEF) and then decays exponentially. This decay signifies the combined effects of reduced ΔP and increased small airway resistance (i.e., diminished radial traction) as the alveolar volume decreases [20, 21]. In the latter part of exhalation, the flow becomes more linear. In certain pathological states, as in patients with small airway obstruction, such as COPD, this reflects the imposed resistance of the artificial airway, primarily the ETT [22–24].

In pulmonary disease, the shape of the V’/V curve varies. This reflects the different severity levels and the uneven distribution of lung compartments with differing resistances, volumes, and elastic recoil properties. In addition, these factors are further altered by the resistances introduced by the artificial airway and ventilator circuit**.** Thus, using a first-order linear model fails to capture the complexity of various lung pathologies [5].

Early physiological studies of passive lung emptying in paralyzed patients derived RC_EXP_ from specific sections of the V’/V curve and the corresponding V_T_. This section of the V’/V curve is significantly transformed from primarily exponential to linear flow decay by evaluating the difference in flow between two-timepoints and their corresponding V_T_, as proposed by Aerts (Eq. 4), Lourens (Eq. 5), and others [9–12, 22, 25]:

4 $${RC}_{EXP}=\frac{{0.5 \cdot V}_{T}}{{{{\varvec{V}}}^{\mathbf{^{\prime}}}}_{50}- {{{\varvec{V}}}^{\mathbf{^{\prime}}}}_{\text{END}-\text{EXP}}}$$

5 $${RC}_{EXP}=\frac{{0.75 \cdot V}_{T}}{{{{\varvec{V}}}^{\mathbf{^{\prime}}}}_{75}- {{{\varvec{V}}}^{\mathbf{^{\prime}}}}_{\text{END}-\text{EXP}}}$$

**Fig. 1:**
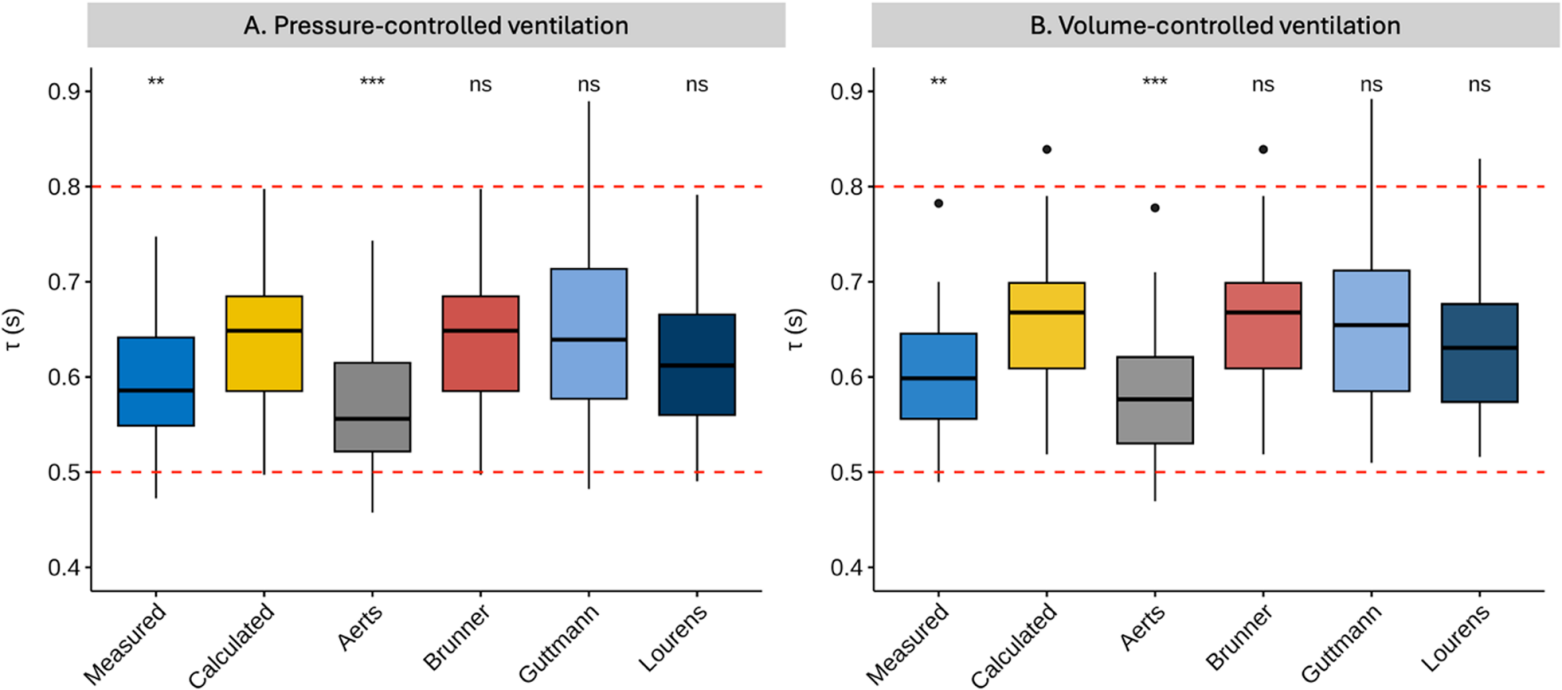
Physiological values of RC_EXP_ in passive, healthy postoperative patients under pressure-controlled ventilation (PCV) and volume-controlled ventilation (VCV) using different methods to obtain RC_EXP_. The upper and lower limits of the reference range are displayed as dashed red lines [14]

RC_EXP_ can also be measured directly from the start of exhalation until 63% of V_T_ has been exhaled to obtain the first RC_EXP_ [14–16, 26–28]. The advantage of such measurement compared to derived or calculated time constants is the ability to measure consecutive RC_EXP_ (i.e., second, third, etc.) and, therefore, independently assess the physical characteristics of nearly whole (i.e., 95% V_T_) of the V’/V curve without needing to use “slicing” method and assess the time constant for each slice [14]. Reference ranges for RC_EXP_ using various methods in postoperative patients are shown in Fig. 1.

With the advancement of electric impedance tomography and high-resolution computed tomography, it is also possible to detect regional RC_EXP_ differences across the lungs [29, 30]. However, this aspect is beyond the scope of this review. Table 3 outlines the pros and cons of each method for determining RC_EXP_ and its bedside application. Table 4 summarizes clinical studies with corresponding RC_EXP_ values across different pathologies in adult human subjects.

**Table 3:** Pros and Cons of different methods to determine RC_EXP_ and its clinical application at bedside

RC_EXP_ method	Pros	Cons	Bedside
Measured (RC_EXP_ = 63% of V_T_) [15]	Directly measured from expiratory flow Includes early portion of exhalation Does not assume single compartmental model	Not shown on most critical care ventilators	Displayed on some ventilators
Calculated (RC_EXP_ = V_T_/PEF) [19]	Easily derived at bedside Considers the whole V_T_	Assumes single compartmental model with linear flow decay	Obtained by hand calculation
Calculated (RC_EXP_ = R_RS_ * C_RS_) [7]	Can be obtained at bedside	Assumes single compartmental model with linear flow decay Requires end-inspiratory and end-expiratory hold maneuvers in paralyzed or deeply sedated patients	Hold maneuvers are required to obtain static C_RS_ and R_RS_
Derived from V’/V curve [10, 22, 25]	Usually displayed on breath-by-breath basis	Assumes single compartmental model	Displayed on some ventilators
Slicing method [6]	Assesses whole exhalation after PEF Obtains the total TC as average from multiple compartments	Under experimental settings	Not available in routine care

*RC*_*EXP*_ expiratory time constant, *V*_*T*_ tidal volume, *PEF* peak expiratory flow, *C*_*RS*_ respiratory system compliance, *R*_*RS*_ respiratory system resistance

**Table 4:** Studies involving human subjects with various lung pathologies and their corresponding RC_EXP_

Author	Year	Pathology	N	Method to obtain RC_EXP_	RC_EXP_ (s)
Guttmann et al. [6]	1995	ARDS	12	Slope of the V’/V curve for each slice	PEEP 11: **0.69** ± 0.2
Kondili et al. [31]	2002	ARDS	10	Regression analysis of V’/V curve for each slice	PEEP 0: **0.82** PEEP 5: **0.77** PEEP 10: **0.74** PEEP 15: **0.68**
Kondili et al. [23]	2004	COPD	10	Regression analysis of V’/V curve for each slice	Early exhalation: **1.19** Late Exhalation: **3.75**
Aerts et al. [25]	1999	COPD Non-COPD	27 12	Slope of the last 50% of V’/V curve	COPD: **3.9** ± 2.2 Non-COPD: **0.8** ± 0.5
Al-Rawas [32]	2013	ARF	92	V_T_/PEF	VC-CMV: **0.58** ± 0.20 VC-SIMV: **0.54** ± 0.27 VC + : **0.66** ± 0.27 PSV: **0.58** ± 0.18
Arnal [10]	2008	Various	243	LSF/75% expiration	Normal: **0.78** (0.64–0.91) ARDS: **0.51** (0.42–0.64) COPD: **1.0** (0.77–1.31)
Arnal [11]	2012	ARF	50	LSF/75% expiration	ASV: **0.7** (0.6–0.8)
Arnal [12]	2013	Various	100	LSF/75% expiration	Normal: **0.61** (0.50–0.75) ARDS: **0.50** (0.43–0.58) COPD: **0.91** (0.64–1.22)
Arnal [9]	2018	Various	359	LSF/75% expiration	Normal: **0.6** (0.5–0.7) ARDS: **0.5** (0.4–0.6) COPD: **1.1** (0.7–2.1)
Belliato [2]	2004	Various	21	LSF/75% expiration	Normal: **0.72** ± 0.14 Restrictive: **0.47** ± 0.14 COPD: **2.21** ± 1.35
Eghtedari [18]	2021	COVID-19 ARDS	60	Product of C_RS_ * R_RS_	Weaned: **0.67** ± 0.23 Died: **0.47** ± 0.19
Iotti [13]	2010	Various	88	LSF/75% expiration	Healthy: **0.71** ± 0.17 Restrictive: **0.50** ± 0.15 Obstructive: **1.08** ± 0.51
Lourens et al. [22]	2000	COPD Other	38	Slope of the last 75% of V’/V curve	Moderate COPD: **0.79–1.47** Severe COPD: **0.85–4.22** Other**: 0.63–1.06**
Lourens et al. [33]	2002	COPD, Non-COPD	20	Fuzzy clustering	COPD: **2.6** ± 1.2 Non-COPD: **0.88** ± 0.2
Dall’Ava-Santucci [28]	1992	Healthy Various ARF	16	63% of V_T_	Healthy: **0.73** ± 0.29 Various AFR: **0.47–6.62**
Depta et al. [15]	2022	COVID-19 ARDS	16	63% of V_T_	PEEP 0: **0.57** (0.47, 0.66) PEEP 5: **0.58** (0.49, 0.68) PEEP 10: **0.61** (0.51, 0.68) PEEP 15: **0.52** (0.43, 0.60)
Depta et al. [16]	2023	Various	56	63% of V_T_	Normal: **0.63** (0.59–0.68) Obstructive: **1.02** (0.96–1.08)
Depta et al. [26]	2023	COVID-19 ARDS	10	63% of V_T_	PEEP 5: **0.63** (0.45–0.66) PEEP 10: **0.67** (0.55–0.76) PEEP 15: **0.67** (0.49–0.72) PEEP 20: **0.62** (0.40–0.69)
Depta et al. [14]	2024	Postoperative patients	30	63%, of V_T_	PCV: **0.59** (0.57–0.62) VCV: **0.60** (0.58–0.63)

The mean ± SD or RC_EXP_ range is provided for each group of subjects. The studies are divided based on the method of RC_EXP_ calculation. *LSF* least square fitting, *V’/V curve* flow/volume curve, *COPD* chronic obstructive pulmonary disease, *ARDS* acute respiratory distress syndrome, *ARF* acute respiratory failure, *COVID-19* Coronavirus Disease 2019, *PEEP* positive end-expiratory pressure, *PCV* pressure-controlled ventilation, *VCV* volume-controlled ventilation, *ASV* assist/support ventilation, *VC-CMV* volume-controlled continuous mandatory ventilation, *VC-SIMV* volume-controlled synchronized intermittent mandatory ventilation, *PSV* pressure support ventilation

### The RC_EXP_ inequalities and non-linear modeling

Theoretical modeling provides a basic insight into volume distribution across the compartments in the non-homogenous lung. Such models comprise more than one theoretical compartment. In their study, Chatburn et al. aimed to determine major factors influencing V_T_ distribution and concluded that RC_EXP_ was the primary factor determining V_T_ distribution between the compartments [34]. Most pathologies requiring mechanical ventilation in clinical care encounter RC_EXP_ inequalities, resulting in heterogeneous V_T_ distribution [29, 35]. To counteract these effects, modulation of V_T_ and PEEP—both major determinants of RC_EXP_—help equalize the homogeneity to redistribute V_T_ across the lung, as elucidated further in the text.

The nonlinear methods for studying RC_EXP_ measure its values throughout the exhalation to examine regional volume distribution and flow heterogeneity during tidal ventilation (i.e., the viscoelastic, resistive, and elastic properties of both the lung and chest wall) [24, 36]. This was achieved by dividing the expired V_T_ into five equal-volume “slices” (hereafter referred to as compartments) [6, 23]. In this method, expired flow and volume are measured from an inflection point (i.e., the point of maximum slope following PEF on the V’/V curve). The rationale for doing so is that [1] RC_EXP_ can only be calculated when the V’/V curve becomes more linear and [2] eliminate mechanical artifacts associated with the opening of the expiratory valve.

Parsing the V’/V curve into equal volume compartments facilitates the identification and characteristics of “fast” and “slow” (i.e., flow-limited) lung compartments. In doing so, RC_EXP_ values can be calculated for each segment, as well as the mean RC_EXP_ for the entire cycle [6, 31]. Multiple studies have evaluated the patterns of lung emptying in two common clinical scenarios—chronic obstructive pulmonary disease (COPD) and acute respiratory distress syndrome (ARDS).

### Characteristics of RC_EXP_ across volume compartments in COPD

Important findings regarding the effect of mechanical ventilation on RC_EXP_ were described by Kondili et al. [23]. Based on their data at zero positive end-expiratory pressure (ZEEP), total RC_EXP_ (i.e., including imposed resistance) increased during exhalation, with mean values τ ranging from 1.19s during the initial phase of expiration to 3.75s in the terminal phase: an increase of 2.56s (average change of 0.64s per volume compartment). When imposed mechanical resistance was removed as a factor, RC_EXP_ of the respiratory system remained virtually unchanged. This is the result of expiratory flow limitation (EFL) caused by small airway collapse, making imposed resistances negligible in this patient population.

**Fig. 2:**
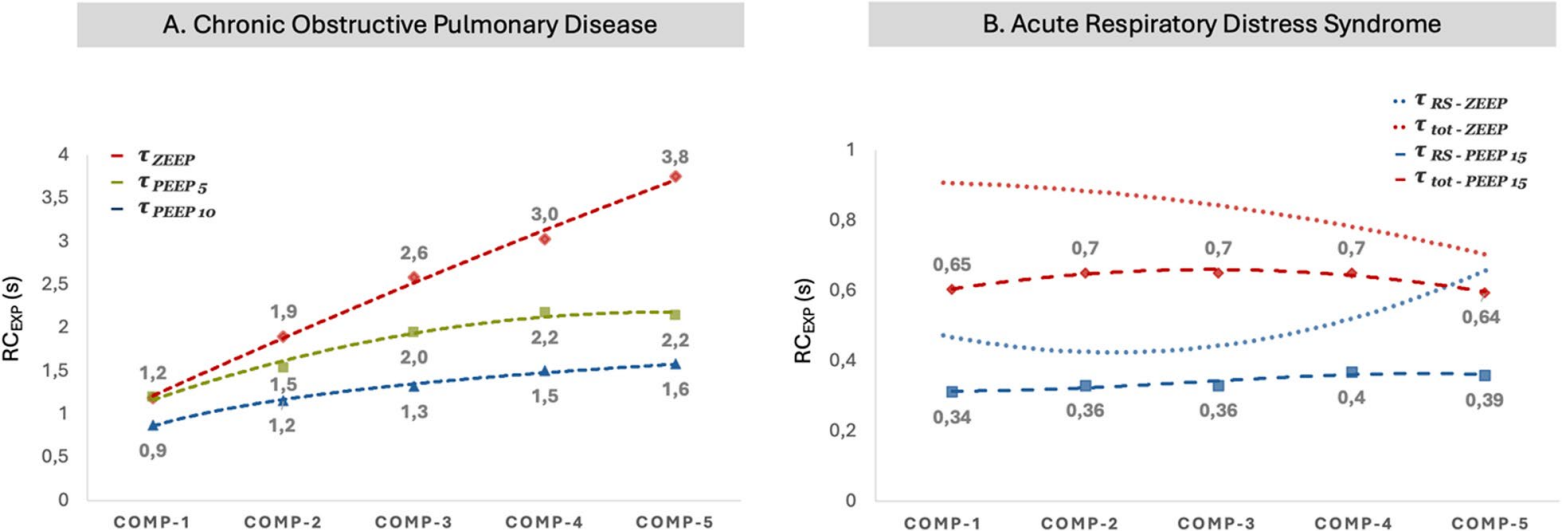
Effects of PEEP on RC_EXP_ in terms of compartments in **A** patients with COPD—increasing PEEP levels reduces the heterogeneity of regional lung emptying and thus promotes exhalation. **B** patients with ARDS—effect of ZEEP (dotted lines) on τ of the total respiratory system including artificial airways (τ tot) and τ of the respiratory system alone (τ RS) and PEEP 15 cmH_2_O (dashed lines). COMP-1–5—compartments 1–5, ZEEP—zero end-expiratory pressure, PEEP—positive end-expiratory pressure, COPD—chronic obstructive pulmonary disease. Figures were reconstructed from the original study by Kondili et al. [23] (**A**), and Guttmann et al. [6] (**B**)

PEEP of 5 and 10 cmH_2_O had a noticeable effect on RC_EXP_, blunting the slope of the RC_EXP_ curve between compartments. Specifically, at a PEEP of 5 cmH_2_O, the average *τ* decreased by 0.36s for compartments 2–4 (19–24%), with the greatest impact observed in compartment 5, where *τ* decreased by 1.6s (43%). PEEP of 10 cmH_2_O produced substantially greater reductions in RC_EXP_ as the average *τ* decreased by 0.7s to 1.53s for compartments 2–5 (39–54%). Again, PEEP had its most pronounced influence on compartments 4 and 5, with an average reduction in *τ* of 2.17s (58%). In other words, PEEP reduced the heterogeneity of regional lung emptying (Fig. 2A).

Lourens et al. studied RC_EXP_ by comparing subjects with moderate and severe COPD under ZEEP conditions [22]. RC_EXP_ was measured between compartments at 100%, 75%, 50%, and 25% of expired V_T_. When using the V’/V technique, RC_EXP_ of the fast compartment (100–76% incomplete exhalation) was indistinguishable between severe and moderate COPD (*τ *of 0.85 and 0.79s, respectively). In contrast, pronounced differences were found between severe and moderate cohorts in the slower compartments (75–51%, 50–26%, and 25–0% incomplete exhalation), which *τ* increased from 0.85s in the fast compartment to 2.84s and 3.42s and 4.2s, respectively, in severe COPD (almost fivefold total increase in RC_EXP_). In contrast, they found milder increases in RC_EXP_ among moderate COPD subjects, wherein values of *τ* increased from an initial value of 0.79s in the early compartment to 1.05, 1.25, and 1.47s, respectively, in the compartments towards the end of exhalation (almost twofold total increase in RC_EXP_).

Thus, an abrupt rise in RC_EXP_ in severe COPD occurring once expired volume reached the 75% (incomplete) threshold at ZEEP signifies marked deterioration of peripheral airway conductance and the need for PEEP to support expiration. PEEP also would be indicated in moderate COPD, although it is reasonable to predict the impact on pulmonary mechanics and/or gas exchange would likely be less pronounced.

Finally, the most salient clinical finding was that 3τ measured at V’/V 75% (incomplete expiration) approximated the actual time for complete exhalation within 0.4 ± 2.4s. It was also used to assess the severity of COPD [22]. This reflects that in COPD patients, airway resistance becomes the major limitation to exhaled flow.

### Characteristics of RC_EXP_ across volume compartments in ARDS

Guttmann et al. were the first to study RC_EXP_ in ARDS using the V’/V method. They reported a τ of 0.69 ± 0.22s whose values differed marginally across expired volume compartments [6] The explanation of this finding was discovered when investigators analyzed total airway resistance into its imposed and respiratory system subcomponents. In ARDS, the resistive characteristics of the lung and chest wall contributions accounted for up to 50% of RC_EXP_. Although it may seem that RC_EXP_ was within the normal range (i.e., 0.69s), it must be stressed that the mean V_T_ used was 989 ml, which confirms that V_T_ is one of the major determinants of RC_EXP_. This is also confirmed by other studies in ARDS patients who reported recruitable lungs, higher V_T,_ and normal or even prolonged RC_EXP_ [15, 26].

Kondili et al. [31] studied the effects of PEEP on RC_EXP_ and they also examined the effects of imposed mechanical resistance. Their RC_EXP_ data was presented as both total its respiratory system subcomponent (RC_EXP_–RS). Across all volume compartments, the mean values of τ for total RC_EXP_ exceeded those of RC_EXP_–RS: τ of 0.82s vs. 0.61s (ZEEP), 0.77s vs. 0.52s (PEEP of 5 cmH_2_O), 0.74s vs.0.57s (PEEP of 10 cmH_2_O) and 0.68s vs. 0.55s (PEEP of 15 cmH_2_O). Thus, the lungs and chest wall accounted for 52 to 61% of total RC_EXP_ and were somewhat higher than those reported by Guttman and colleagues.

The effects of PEEP on total RC_EXP_ and RC_EXP_–RS were more complex and informative when assessed by its impact on fast and slow lung compartments. In contrast to the inconsistent relationship between total RC_EXP_ and estimated RC_EXP_–RS that were observed among COPD subjects in response to PEEP, the τ-compartment patterns found in ARDS mirrored one another despite their marked separation in values of τ [31]. Consistent with the findings of Guttmann and colleagues [6], PEEP largely homogenized values of τ across compartments (Fig. 2B). This contrasted with total RC_EXP_ and RC_EXP_–RS under ZEEP conditions, wherein fast vs. show compartments were salient (albeit not as stark as that found with ZEEP in their COPD subjects).

Chelucci et al. [17] used respiratory inductive plethysmography to measure thoracic volume rather than gas volume during tidal ventilation. They observed distinct fast and slow lung compartments, wherein RC_EXP_ of the slow compartment was 11–13-fold longer than the fast compartment, irrespective of V_T_ size (10 vs. 5 mL/kg) and PEEP (13 cmH_2_O) vs. ZEEP. In the presence of PEEP, the fast compartment had mean τ values 16% greater at 10 vs. 5 mL/kg (0.58 vs. 0.50s, respectively), whereas corresponding slow compartment τ values were unchanged (6.44 vs. 6.46s, respectively). Removal of PEEP resulted in marked reductions in RC_EXP_ in both the fast (40% decrease from τ of 0.58 to 0.35s) and slow (27% decrease from τ of 6.44 to 4.67s) compartments. The impact of PEEP on RC_EXP_ in ARDS suggests it counters the compressive hydrostatic or elastic forces acting upon open lung units with negligible peripheral obstruction. Without additional information (i.e., volumetric capnography phase characteristics), the interpretation of slow compartment changes RC_EXP_ remains problematic.

Generalizing the characteristics of RC_EXP_ in ARDS, and particularly the impact of PEEP, should be done with caution. It should consider that ARDS evolves through overlapping phases of acute lung injury that alter both lung morphology and mechanics, as well as the impact of age (i.e., age-related increases in closing volume) as well as alterations in the chest wall components (i.e., both the thoracic cage and abdominal wall) over time. In addition, the expiratory resistance ventilator circuit resides predominantly in the expiratory manifold, which contains the valve, pressure, and flow sensors of varying configurations and control characteristics. All of these, to some extent, modulate expiratory flow at different points in exhalation. Without specific knowledge as to their functioning (the sophistication of which changes over time within and between brands), a degree of uncertainty will remain about the characteristics and magnitude of total RC_EXP_ and its actual relationship to RC_EXP_–RS.

**Fig. 3:**
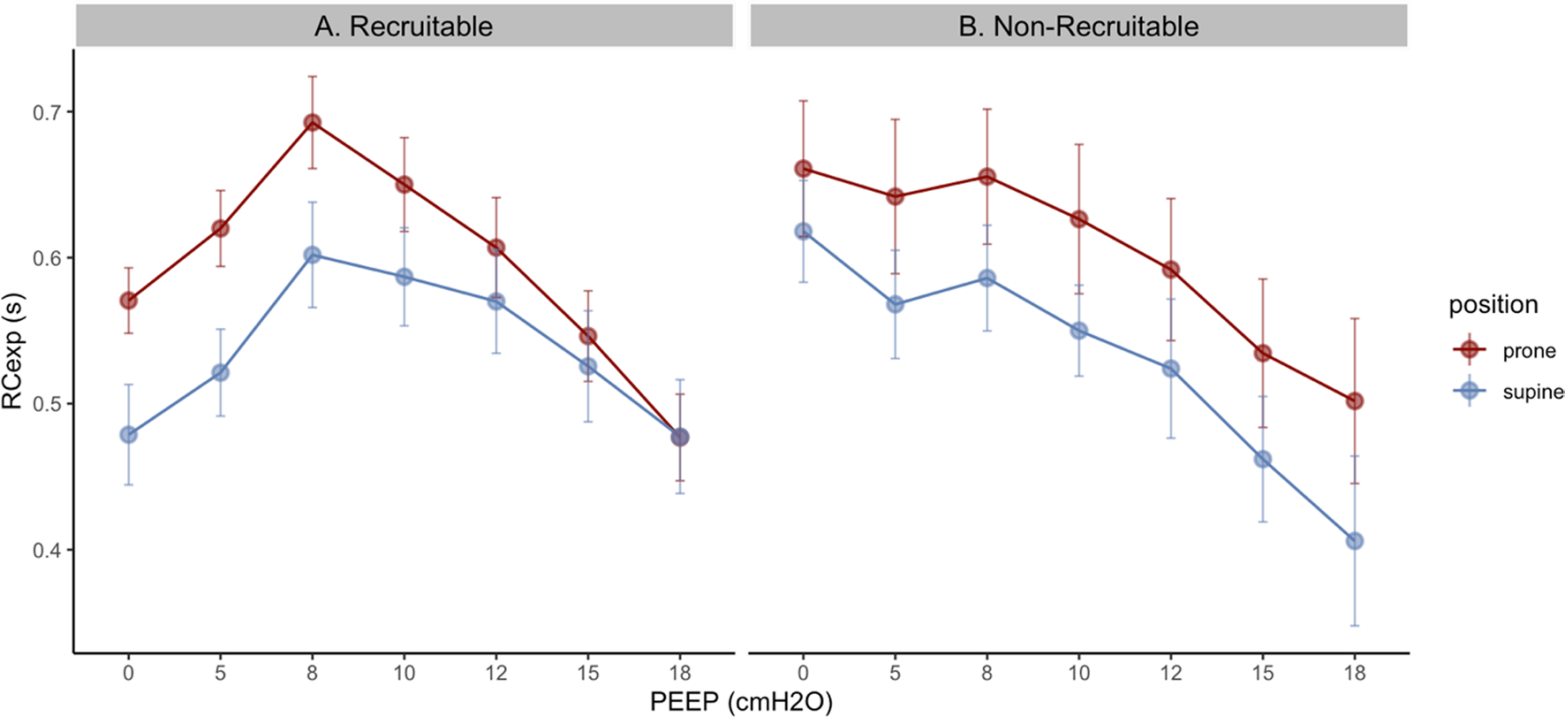
PEEP titration and recruitability patterns in COVID-19 patients with moderate to severe ARDS in the prone and supine positions. Mean values of RC_EXP_ are displayed at each PEEP in both positions. Figures were reconstructed from Depta et al. [15]

A physiologic study on COVID-19-related ARDS assessed the impact of PEEP titration on RC_EXP_ in 16 passive patients ventilated with a fixed pressure control of 14 cmH_2_O [15]. The research aimed to identify optimal PEEP levels similar to the best C_RS_ by focusing on maximum RC_EXP_. Findings showed that increased RC_EXP_ (assuming recruitment) correlated with optimal C_RS_. The authors concluded that RC_EXP_ is a potential tool for determining PEEP levels in both supine (RC_EXP_ of 0.59s at PEEP 8 cmH_2_O) and prone positions (RC_EXP_ of 0.67s at PEEP 8 cmH_2_O) (Fig. 3). Moreover, the study identified recruitable vs. non-recruitable lungs based on RC_EXP_ trends during the ascending PEEP trial. This is also supported by Eghtedari et al., who found that patients with shorter RC_EXP_ (0.47s) had higher ARDS mortality than those who were successfully weaned from mechanical ventilation (RC_EXP_ of 0.67s) [18]. In their study, the resulting low RC_EXP_ in patients that died (0.47 s) was more due to lower C_RS_ (37 vs. 75 mL/cmH_2_O) compared to an increase in R_RS_ (14 vs. 9 cmH_2_O/L/s). [30].

Similarly, a study of 10 COVID-19-related ARDS cases found a significant correlation between RC_EXP_ and the dead space to tidal volume fraction (V_D_/V_T_) during ascending PEEP trials (r =  − 0.72). The highest RC_EXP_ was observed at 12 cm H_2_O PEEP, while the lowest V_D_/V_T_ was at 10 cmH_2_O, and the highest C_RS_ occurred at 15 cmH_2_O. It remains unclear if exhalation dynamics (RC_EXP_) reflect global ventilation/perfusion responses [26]. Although setting PEEP based on RC_EXP_ (i.e., dynamic changes of both C_RS_ and R_RS_) is promising due to its physiological rationale, this has not been investigated further, and its implications in clinical care are currently limited [15].

To conclude, the relationship between PEEP and RC_EXP_ is essential for understanding changes in lung mechanics, particularly regarding lung recruitability (i.e., recruitment-induced changes in V_T_, which influence RC_EXP_). In cases of heterogeneous lung disease, PEEP plays a significant role in enhancing the distribution of V_T_ across various RC_EXP_ regions. However, using excessively high PEEP can lead to a reduced RC_EXP_, characterized by rapid flow due to excessive stretching and lung overdistension (Fig. 3).

### Clinical significance of RC_EXP_

The clinical interest in RC_EXP_ became a focal point for research in the early 1980s after the elucidation of the phenomena of dynamic hyperinflation, incomplete exhalation, and intrinsic PEEP became a priority [37, 38]. Compared to commonly used variables of lung mechanics (i.e., C_RS_, ΔP, or plateau pressures), RC_EXP_ may have been undervalued clinically. As a function of V_T_ and PEEP, RC_EXP_ allows for basic ventilator adjustments. It can be measured during passive exhalation, reducing bias compared to traditional inspiratory metrics obtained during clinician-controlled inspiration, and can serve several purposes.

Some mechanical ventilators enable continuous RC_EXP_ monitoring on a breath-to-breath basis (or, as an average, across multiple previous breaths) without the need to perform inspiratory and expiratory hold maneuvers that are not possible in spontaneously breathing patients. Apart from obstructive diseases whose hallmark is increased airway resistance, RC_EXP_ can also be prolonged in patients with recruitable lungs with high V_T_, outside of the range that is currently considered protective [39].

As described previously, along with conventional variables, RC_EXP_ can help in adjusting PEEP levels. If recruitability is confirmed and increased V_T_ is observed with constant ΔP, RC_EXP_ will also increase. Therefore, optimized RC_EXP_ can be viewed as a sign of a positive response to recruitment [15]. In contrast to excessively high PEEP, RC_EXP_ will be short as overdistension will cause low compliance and fast backflow (i.e., recoil) from the lungs. Feasibility studies in ARDS patients have explored the utility of RC_EXP_ to determine optimal PEEP levels, demonstrating its ability to assess recruitability in both prone and supine positions [15]. This may aid PEEP selection in addition to conventional methods used in routine clinical care [40], but is currently a subject of further research.

Incomplete exhalation (i.e., EFL) has traditionally been a hallmark in COPD patients. However, multiple studies have shown that even in patients without COPD, the prevalence of EFL is up to 30% in the general ICU population [41, 42]. In these patients, there will be incomplete exhalation on the flow waveform (i.e., flow not returning to zero) along with prolonged RC_EXP_. In such circumstances, increasing PEEP can partially prevent small airway collapse and promote more complete exhalation [23, 29]. In both groups of patients with heterogeneously affected lungs, RC_EXP_ became more equalized across compartments, and thus, PEEP reduced the heterogeneity of regional lung emptying.

Adjusting the RR based on RC_EXP_ to prevent dynamic hyperinflation and intrinsic PEEP seems rational. However, expiratory time (and thus RR) cannot be predicted from the single value of RC_EXP,_ which usually considers only some portion of the V’/V curve or considers the whole respiratory system as linear function. A report in a heterogeneous ICU population revealed that clinicians set RR on the ventilator exceeded that predicted by RC_EXP_ in the majority of patients [16]. However, the study did not account for the fact that RC_EXP_ cannot be extrapolated from a single RC_EXP_ value, as proven later [14] As a result, RC_EXP_ can help optimize but cannot accurately predict the RR based on a single RC_EXP_ value.

**Fig. 4:**
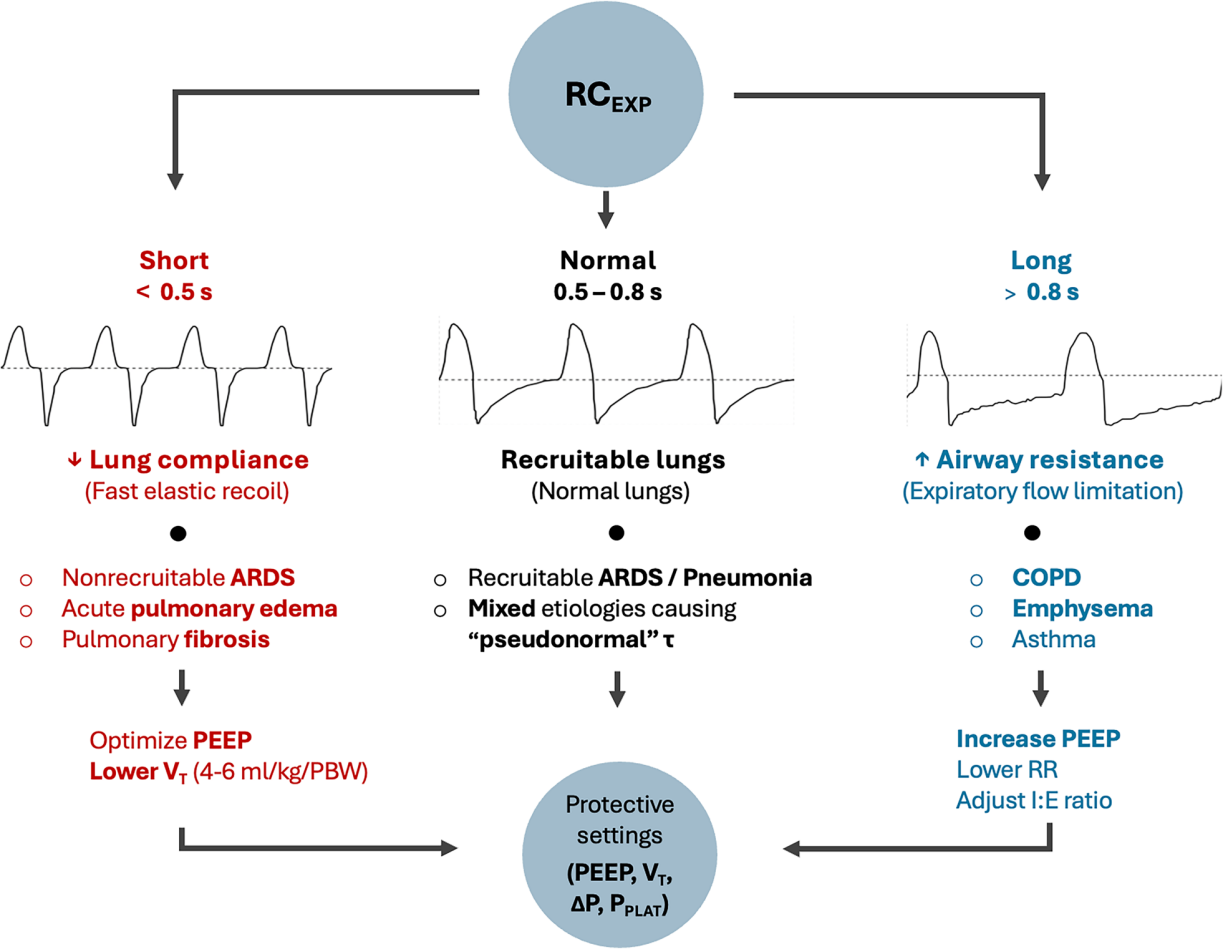
Common clinical scenarios and ventilator adjustments based on RC_EXP_. **A**. Short RC_EXP_ typical of acute pulmonary edema and "stiff" lungs, such as nonrecruitable ARDS and pulmonary fibrosis, **B**. Normal RC_EXP_ typical for recruitable lung (i.e. ARDS, pneumonia) including mixed pathologies creating "pseudonormal" RC_EXP_, **C**. Long RC_EXP_ associated with expiratory flow limitation due to high airway resistance

Along with the directly observed shape of the flow waveform, RC_EXP_ can complement exhalation patterns (i.e., the graphical shape of the expiratory flow curve). In acutely admitted patients without known prior lung disease, revealing variations from expected normal values may facilitate timely diagnosis or changing respiratory mechanics due to improvement or worsening. Figure 4 shows common clinical scenarios and ventilator adjustments based on  RC_EXP_.

Finally, RC_EXP_ allows real-time calculations of plateau pressure, C_RS_, and R_RS_ without the need for an end-inspiratory pause, which can improve patient assessment without disrupting ventilation. A study by Al-Rawas [32] explored the feasibility of RC_EXP_ in obtaining common variables of respiratory mechanics in patients with acute respiratory failure at various ventilatory modes. It found that RC_EXP_-modified equations for C_RS_, R_RS_, and plateau pressure yielded nearly the same results comparable to the conventional end-inspiratory and end-expiratory hold maneuvers and allow for continuous assessment of lung mechanics.

### RC_EXP_ modulation at bedside

In acute lung injury with recruitment potential, RC_EXP_ usually increases with PEEP, reflecting recruited lung volume and overall slower passive elastic recoil. Conversely, overdistension from excessive PEEP will decrease RC_EXP_ due to low compliance. In non-recruitable lungs, such as acute pulmonary edema or stiff ARDS, RC_EXP_ will be shorter with increasing PEEP due to limited alveolar recruitment and increased regional overdistension. PEEP titration helps determine optimal recruitability and PEEP levels, balancing protective V_T_ and/or ΔP to achieve the highest RC_EXP_, which has been suggested to represent the best tradeoff between recruitment and overdistension [15]. Increased V_T_ generally prolongs RC_EXP_, while decreased V_T_ has the opposite effect. On the other hand, obstructive lung diseases like COPD, show prolonged RC_EXP_ due to high C_RS_ and R_RS_. In these cases, increasing PEEP can help counteract intrinsic airway resistance and promote exhalation. However, PEEP should be set carefully along with the gas exchange, respiratory mechanics, and hemodynamics.

Change in RC_EXP_ is also observed as clinical conditions evolve, such as prolonging RC_EXP_ with resolving pulmonary edema or pseudo-normal RC_EXP_ in mixed respiratory conditions, such as COPD with concomitant pneumonia.

RC_EXP_ should not be normalized to fit the usual reference range (i.e., 0.5–0.8 s) in all patients, as this is unachievable in many clinical scenarios (i.e., as high minute ventilation requirements or the upper range of protective inspiratory pressures, ultraprotective V_T_, etc.). Instead, it should guide adjustments in inspiratory variables to improve overall lung mechanics.

In summary, RC_EXP_ is a dynamic variable that must be interpreted in the context of ventilator settings and evolving pathology over time.

### Limitations and future directions

Assuming a single linear model for calculating RC_EXP_ greatly oversimplifies the complexities of lung emptying. Focusing only on the first (i.e., 63% of V_T_) or the “one” (i.e., a particular segment of the V’/V curve) leaves the rest of the exhalation without numeric expression. Regardless of the methodology, such simplification limits the ability to conduct a more thorough analysis of the entire exhalation. An option to address this limitation may be direct measurement of the consecutive time constants (i.e., τ1, τ2, τ3, etc.) directly from the expiratory flow waveform. Additional research into more representative numerical variables for assessing exhalation as a whole is required.

## Conclusion

RC_EXP_ reflects expiratory lung mechanics in mechanically ventilated patients. It represents the passive elastic recoil of the lung and chest wall, including artificial resistance. As a dynamic variable, RC_EXP_ must be interpreted in the context of underlying respiratory pathology (and its changes over time) and ventilator settings, particularly V_T_ and PEEP. In patients with heterogeneous lung involvement, such as ARDS and COPD, PEEP can promote a more uniform distribution of regional time constants, thereby improving lung emptying and ventilation homogeneity.

## Data Availability

Not applicable.

## Abbreviations

ARDS: Acute respiratory distress syndrome
COPD: Chronic obstructive pulmonary disease
COVID-19: Coronavirus disease 2019
C_RS_: Respiratory system compliance
ETT: Endotracheal tube
PEEP: Positive end-expiratory pressure
PEF: Peak expiratory flow
RC_EXP_: Expiratory time constant
RR: Respiratory rate
R_RS_: Respiratory system resistance
V_T_: Tidal volume
V’/V curve: Flow/volume curve
ZEEP: Zero end-expiratory pressure
