# Correction: Expiratory time constants in mechanically ventilated patients: rethinking the old concept—a narrative review

**DOI:** 10.1186/s40635-025-00757-5

**Published:** 2025-05-06

**Authors:** Filip Depta, Richard H. Kallet, Michael A. Gentile, Elias N. Baedorf Kassis

**Affiliations:** 1 Department of Critical Care, East Slovak Institute for Cardiovascular Diseases and Faculty of Medicine, Pavol Jozef Šafárik University, Košice, Slovakia; 2 Respiratory Care Services, Department of Anesthesia and Perioperative Care, San Francisco General Hospital, San Francisco, CA USA; 3 Department of Anesthesiology, Duke University Medical Center Durham, Durham, NC USA; 4 Beth Israel Deaconess Medical Center and Harvard Medical School, Boston, MA USA

**Correction: Intensive Care Medicine Experimental (2025) 13:40** 10.1186/s40635-025-00745-9

Following publication of the original article [1], it came to the authors' attention that there was an error in Table 1: in the heading row, where it now (correctly) says '"Long RCexp > 0.8 s',  instead of the previous  'Long RCexp > 0.5 s'. The authors thank you for reading this erratum and apologize for any inconvenience caused.
